# Dysmetria and Errors in Predictions: The Role of Internal Forward Model

**DOI:** 10.3390/ijms21186900

**Published:** 2020-09-20

**Authors:** Pierre Cabaraux, Jordi Gandini, Shinji Kakei, Mario Manto, Hiroshi Mitoma, Hirokazu Tanaka

**Affiliations:** 1 Service de Neurologie, Médiathèque Jean Jacquy, CHU-Charleroi, 6000 Charleroi, Belgium; pcabaraux@gmail.com (P.C.); jordig85@gmail.com (J.G.); mmanto@ulb.ac.be (M.M.); 2 Laboratory for Movement Disorders, Tokyo Metropolitan Institute of Medical Science, Tokyo 156-8506, Japan; kakei-sj@igakuken.or.jp; 3 Service des Neurosciences, University of Mons, 7000 Mons, Belgium; 4 Department of Medical Education, Tokyo Medical University, Tokyo 160-0023, Japan; 5 Faculty of Information Technology, Tokyo City University, Tokyo 158-8557, Japan; hirokazu@jaist.ac.jp

**Keywords:** cerebellum, cerebellar ataxias, dysmetria, prediction, internal forward model

## Abstract

The terminology of cerebellar dysmetria embraces a ubiquitous symptom in motor deficits, oculomotor symptoms, and cognitive/emotional symptoms occurring in cerebellar ataxias. Patients with episodic ataxia exhibit recurrent episodes of ataxia, including motor dysmetria. Despite the consensus that cerebellar dysmetria is a cardinal symptom, there is still no agreement on its pathophysiological mechanisms to date since its first clinical description by Babinski. We argue that impairment in the predictive computation for voluntary movements explains a range of characteristics accompanied by dysmetria. Within this framework, the cerebellum acquires and maintains an internal forward model, which predicts current and future states of the body by integrating an estimate of the previous state and a given efference copy of motor commands. Two of our recent studies experimentally support the internal-forward-model hypothesis of the cerebellar circuitry. First, the cerebellar outputs (firing rates of dentate nucleus cells) contain predictive information for the future cerebellar inputs (firing rates of mossy fibers). Second, a component of movement kinematics is predictive for target motions in control subjects. In cerebellar patients, the predictive component lags behind a target motion and is compensated with a feedback component. Furthermore, a clinical analysis has examined kinematic and electromyography (EMG) features using a task of elbow flexion goal-directed movements, which mimics the finger-to-nose test. Consistent with the hypothesis of the internal forward model, the predictive activations in the triceps muscles are impaired, and the impaired predictive activations result in hypermetria (overshoot). Dysmetria stems from deficits in the predictive computation of the internal forward model in the cerebellum. Errors in this fundamental mechanism result in undershoot (hypometria) and overshoot during voluntary motor actions. The predictive computation of the forward model affords error-based motor learning, coordination of multiple degrees of freedom, and adequate timing of muscle activities. Both the timing and synergy theory fit with the internal forward model, microzones being the elemental computational unit, and the anatomical organization of converging inputs to the Purkinje neurons providing them the unique property of a perceptron in the brain. We propose that motor dysmetria observed in attacks of ataxia occurs as a result of impaired predictive computation of the internal forward model in the cerebellum.

## 1. Introduction

Lesions of the cerebellum elicit inaccuracy and clumsiness in limb movements and instability in posture and gait and ocular saccades, collectively termed as “ataxia”, the diseases being gathered under the umbrella of cerebellar ataxias (CAs) [1]. The term “ataxia” originates from Bouillaud (1846), who used this term to describe a failure of coordination in voluntary movements [2]. In addition to motor control, cerebellar dysfunction also generates similar deficits in executive functions, linguistic processing, spatial cognition, and affect regulation (cerebellar cognitive affective syndrome, also referred to as the Schmahmann syndrome) [3]. Patients with episodic ataxia also exhibit recurrent episodes of ataxia in the form of attacks, including motor dysmetria. Patients typically report episodes of clumsiness and lack of coordination.

Among a wide range of manifestations of cerebellar ataxias, dysmetria is a core symptom in cerebellar motor ataxias [4] and also in cerebellar cognitive affective syndrome (dysmetria of thought) [5]. Babinski first reported dysmetria in 1899 [6]. Its most common form is hypermetria, a movement overshooting the target. For example, in the finger-to-nose test, the index finger initially follows an intended direction but later exceeds the aimed nose and hits the cheek. That movement ends in a few oscillations until the finger finally reaches the nose [2]. Cerebellar patients can also show an undershoot or premature arrest before the target, called hypometria. In some patients, both forms of dysmetria coexist. In others, hypometria follows hypermetria during an aberrant recovery after an acute cerebellar lesion such as a cerebellar stroke [4]. Dysmetria is present proximally and distally both in the upper and lower limbs [4]. Garcin characterized these outstanding features as disturbances in the amplitude of movements [2]. 

Ataxic movements are typically slow and inaccurate and accompanied by excessive movement corrections. Movement deviation becomes more significant for actions performed as fast as possible [4], where patients face difficulty in utilizing a compensatory strategy. Marsden and colleagues examined the effects of visual compensations on dysmetria using a task of free, unrestrained reaching movements of the arm [7]. The spatial path was circuitous in cerebellar patients, rather than straight ones in healthy controls, accompanied by excessive changes in directions during the last part of the movement. Under visual guidance, the patients corrected their moves toward the target. In other words, the vision had no measurable effects on the route taken. Notably, visual adjustments in the end phase of the movement were not regular, resulting in excessive directional changes near the target. They concluded that the ataxic path was “the natural consequence of inappropriate programming of muscle activity” or “the result of proprioceptive feedback incorrectly modifying the central commands” [7]. The clinical observation suggests that a malfunction in the central programming and the peripheral feedback underlies irregular movements observed in dysmetria. 

Recent computational and physiological studies have shown that the cerebellum predicts current and future states of the body and environments by integrating an estimate of the previous state and a given efference copy of motor commands [8,9,10]. This predictive computation is often referred to as an internal forward model, a model of the body and environments causally forward in time. An internal forward model transforms a motor command into a prediction of the sensory consequences of a movement. Interestingly, the ataxic status assumed by Marsden et al., “impaired interaction of periphery feedback with central programming,” coincides with possible dysfunctions induced by an internal forward model. The forward model was initially proposed as a physiological mechanism to compensate for the delay in sensory feedback for stable control of rapid actions. We argue that the predictive computation of the forward model affords error-based motor learning, coordination of multiple degrees of freedom, and appropriate timing of muscle activities. Considering the multifunctioning of the forward model, we propose that various features observed in dysmetria can be traced back to a single origin, that is, an impairment in the internal forward model. Throughout this review, we materialize the view that the central programming referred to by Marsden corresponds to the predictive computation afforded by the internal forward model.

This review will focus on pathomechanisms underlying dysmetria in cerebellar motor ataxias. We will evaluate the hypothesis that dysmetria is a result of impairments in an internal forward model. For this aim, we first review, in Section 2, the characteristics of cerebellar macro- and micro-anatomy as a universal neural substrate for predictive computations. We emphasize that the cerebellum forms closed connections with not only motor-related areas but also the prefrontal cortex and the limbic areas. In Section 3, we elaborate on the theoretical foundations of the internal forward model and its possible computational roles in online prediction, motor learning, and coordinating multiple degrees of freedom and activity timing. In Section 4, we summarize neural discharge characteristics of the cerebellar cells, particularly the Purkinje cells, recorded from behaving animals. The Purkinje cell activities in a functional unit encode motor parameters, which concurs the forward-model computation in the cerebellum. In Section 5, we provide substantial evidence that the cerebellum is a locus for the internal forward model by summarizing two of our recent studies. Finally, in Section 6, we revisit a range of previously reported features of kinematics and muscle activities found in ataxic patients and argue that these features manifest as a result of an impaired computation in the internal forward model. In particular, the traditional views on dysmetria, the synergy- and timing-theories, are explained from the perspective of a predictive forward model.

## 2. Cerebellum and Predictions: Relevant Anatomy

### 2.1. Macroanatomy-Physiological Classification

**Figure 1:**
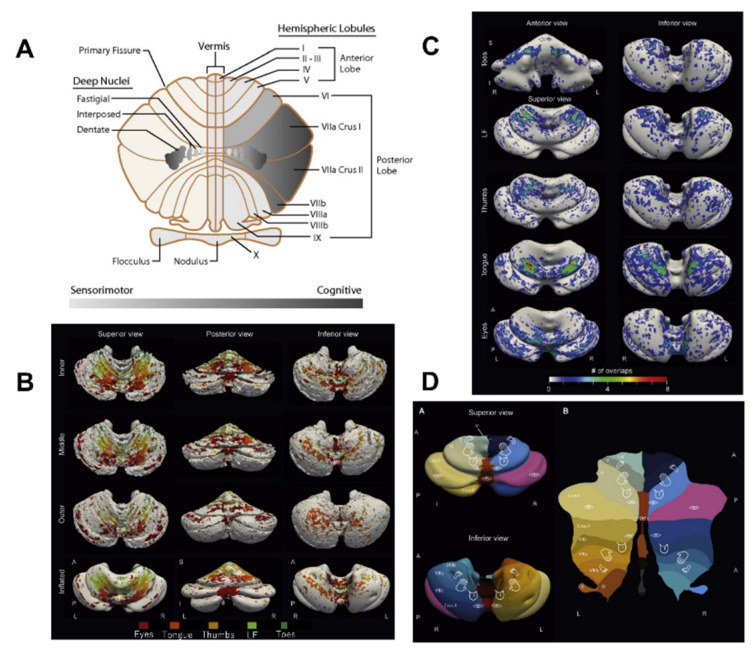
(**A**) scheme of cerebellar sensorimotor and cognitive topography. The cerebellum is divided into 10 lobules (I-X; classification of Larsell). Classically, the cerebellum has been divided into an anterior lobe (lobules I-V), a posterior lobe (lobules VI-IX) and a flocculo-nodular lobe (lobule X). The lateral portions of the cerebellum are particularly prominent in humans. (**B**) Somatomotor maps of the cerebellum obtained in healthy adults. L/R: left-right; A/P: anterior-posterior; S/I: superior-inferior. (**C**) Inflated surface on which the number of overlaps for each body part is mapped. (**D**) Illustration of the somatotopic organization (superior view, inferior view, and flatmap). Figure 1B–D: from [14].

**Figure 2:**
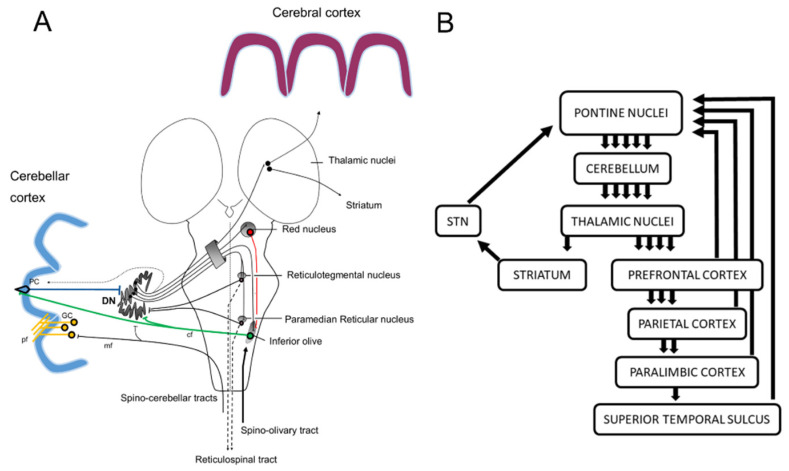
Illustration of the multiple loops between the cerebellum and cerebral cortex. (**A**) A corticonuclear projection from the Purkinje cell (PC) to the dentate nucleus (DN) is shown. The dotted line corresponds to a recurrent collateral ending in the the same sagittal zone at the origin of the PC to DN projection. The DN is involved in dentato-reticular reverberating loops, in the dentato-rubro-olivary triangle (Guillain-Mollaret) and in dentato-thalamo-cortical projections. The climbing fibers from the inferior olive project to both the DN and the PC via climbing fibers (cf). The mossy fibers of the spino-cerebellar tracts target the DN and the granule cells (GC) at the origin of the parallel fibers (pf). The afferents to the DN from the locus coeruleus and the raphe nuclei are not illustrated. Both the spinocerebellar tracts and the trigeminocerebellar tracts (not illustrated) relay information from the limbs and head/face, respectively. (**B**) Multiple loops running in parallel from the cerebellum to the cerebral cortex and to the striatum. The striatum projects to the subthalamic nucleus (STN) which targets pontine nuclei (disynaptic projection from the STN to the cerebellar cortex). The cerebellum receives not only corticopontine afferents from motor/nonmotor areas, but also information from the superior colliculus, the mammillary bodies and projects towards the ventral tegmental area (VTA), the locus coeruleus and the hypothalamus, which are connected with the limbic/paralimbinc regions linked to emotional processing (cerebro-cerebellar-limbic loops, not illustrated). The so-called limbic cerebellum engages mostly midline areas of the cerebellum.

**Figure 3:**
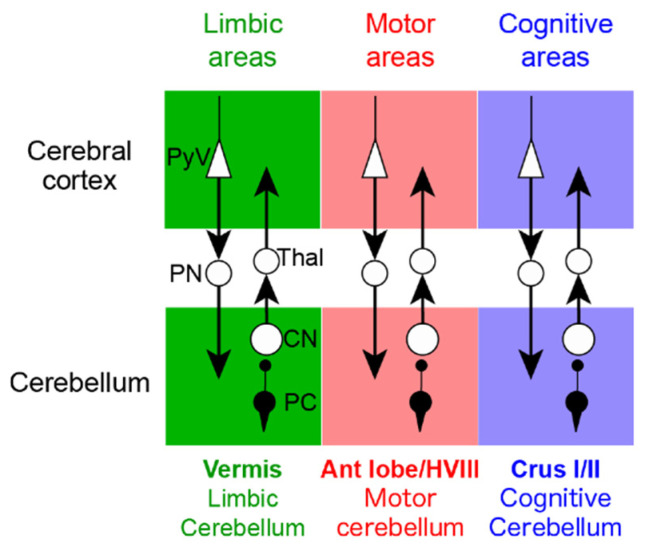
The parallel organization of the cerebro-cerebellar loops. The cerebellum contains anatomically separate and functionally distinct motor and nonmotor domains. CN: cerebellar nuclei cells; PC: Purkinje cells; PN: Pontine nuclei cells; PyV: Layer V Pyramidal cells; Thal: Thalamus.

Several physiological classifications of the cerebellum have been advanced in the last years with the demonstration that the cerebellum contributes to non-motor functions, in addition to the motor functions (Figure 1). The most classical view of the cerebrocerebellar loop is that the cerebellum receives information from various areas of the cerebral cortex, including association areas, and then funnels this information back to the primary motor cortex [11], suggesting that the cerebellum integrates a range of information to guide motor control [12]. It should be noted that the funnel organization of the cerebrocerebellar loop appears to be most compatible with the inverse dynamics model of the cerebellum (see Section 3). However, more recent anatomical studies using transneuronal tracers have challenged this motor-centric view of the cerebellum [12,13] (Figure 2 and Figure 3). In this new view, the cerebrocerebellar loop is divided into several parallel loops [12,13]. Each region of the cerebrocerebellum receives an input from a specific region of the cerebral cortex, and in return, projects back to the corresponding cortical region (Figure 3). Thus, motor, cognitive and limbic functions are spatially separated within the cerebellum, embedded in parallel loops with the corresponding regions of the cerebral cortex (Figure 3). The current anatomical view of the cerebellum with multiple parallel loops allows the concurrent assessment of the visuomotor and cognitive computations. On the other hand, it may not be straightforward to fit the parallel loop organization with the inverse dynamics model of the cerebellum.

The cerebellar cortex is characterized by the homogeneous “crystal-like” organization. Nevertheless, the parallel loop organization mentioned above suggests functional localization of the cerebellar cortex. Indeed, there are two known gradients in the cerebellar cortex: rostrocaudal and mediolateral.

#### 2.1.1. Rostrocaudal Gradient

Anterior cerebellar lobe (lobule I-VI) and lobule VIII relate to sensorimotor regions of the cerebral cortex and participate in motor tasks (Figure 1). Lobule VII exhibits significant connectivity with the parietal and prefrontal cortex (Figure 1A). Lobule IX and X are classified as a non-motor representation and relate to default mode processing or attentional/executive processing [15]. Several structural and functional neuroimaging techniques showed this lobule’s involvement in cognitive tasks [16].

#### 2.1.2. Medio-lateral Gradient

Besides these rostrocaudal compartments, the mediolateral axis separates cerebellar functions. The medial and intermediate cerebellar cortex controls gait and stance, and the intermediate and lateral cerebellar cortex control limb movements [1]. 

In terms of somatomotor maps, recent studies with 7T fMRI confirms that multiple representations of the distal body parts are found in the human cerebellum and organized in an orderly fashion (Figure 1B–D) [14]. The anterior lobe shows the most robust somatotopic organization and bilateral eyes-to-toes gradients follow a medial-posterior to lateral-anterior orientation. These important findings indicate that a lobule-wise parcellation of the cerebellum needs to take into account (1) the multiple representations across several lobules and (2) the distribution of projections from the inferior olive to the cerebellum [14]. 

A recent study also reported functional compartmentalization of the cerebellum for different associative learning. The medial and lateral zones contribute to fear conditioning, whereas the intermediate zone handles eyeblink conditioning [17]. Several authors suggest that the right cerebellum is associated with language and non-motor functions because of its connectivity with the left cerebral hemisphere [16].

### 2.2. Microanatomy of the Cerebellar Cortex

#### 2.2.1. Microzones

**Figure 4:**
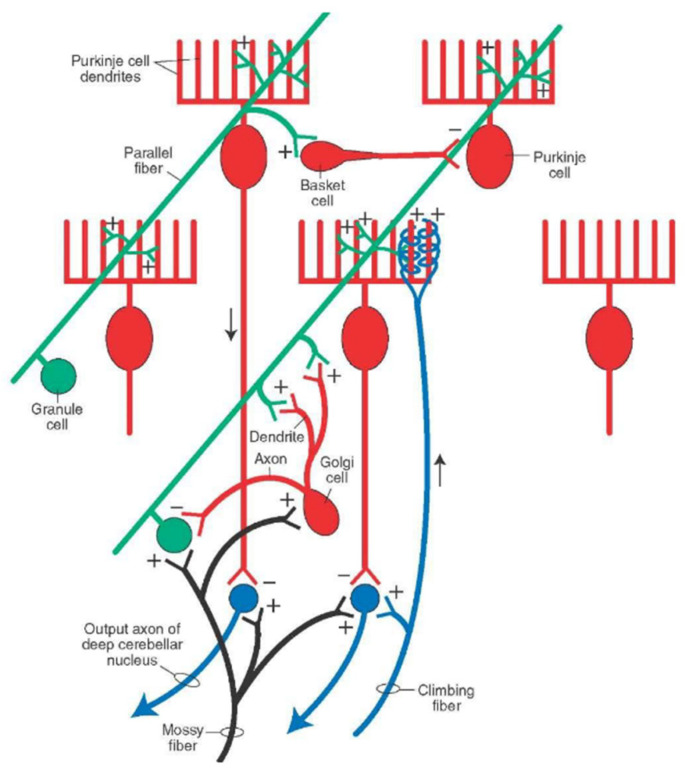
Neural circuit of the cerebellar cortex. The neurons indicated by green or blue are excitatory neurons, whereas the neurons indicated by red are inhibitory neurons.

**Figure 5:**
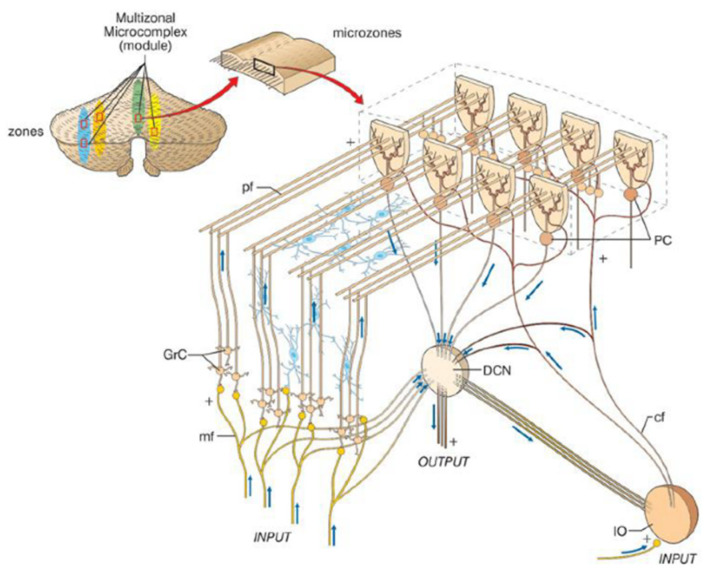
A schematic drawing of microzone, a functional unit in the cerebellar cortex. PC; Purkinje cells, GrC; granule cells, mf; mossy fiber, pf; parallel fiber cf; climbing fiber, DCN; deep cerebellar nucleus, IO; inferior olive nucleus. From: [23] Seeking a unified framework for cerebellar function and dysfunction: from circuit operations to cognition.

The cerebellar cortex receives two major afferents; mossy fibers and climbing fibers (Figure 4). The functional unit of the cerebellar cortex is hypothesized as a microzone, a rostrocaudal strip of the cerebellar cortex (Figure 5) [18]. Purkinje cells (PCs) in a microzone receive climbing fibers from a group of neurons in a restricted area of the inferior olive nucleus, and in turn, these PCs project to a small group of neurons in a deep cerebellar nucleus. This part of the nucleus is assumed to receive collaterals of the same climbing fibers targeting to the microzone, and contain GABAergic neurons that project back to the same area of the inferior olive nucleus. The precise olivo-cortico-nuclear circuitry is assumed to constitute the core circuit of the microzone. A population of PCs in a given microzone are assumed to operate in a functionally similar manner [18]. This type of functional unit is assumed to correspond to the cerebellar microcomplex proposed by Ito [19]. Thus, the olivocerebellar projection provides a basis of the microcomplex. In contrast, mossy fibers give rise to numerous branches to terminate on granule cells (GCs; convergence of 4/1 from mossy fibers to GCs) in a number of microzones with a medio-laterally divergent pattern [20,21]. Axons of GCs (parallel fibers, PFs) run more than several millimeters mediolaterally along the folium, and, as a result, each PC receives numerous PF inputs (~10^4^) in primates [19]. Notably, these characteristic architectures appear suitable to integrate motor efference copy and sensory afferent on single PCs, a necessary substrate for an internal forward model [22].

#### 2.2.2. Cerebellar Modules

Several morphological and electrophysiological studies suggested that the cerebellar microcomplexes are organized into more extended anatomical entities known as cerebellar modules or olivo-cortico-nuclear modules [17,24]. Along the mediolateral axis in each side of the cerebellum, there are twelve longitudinal zones, which have different input-output organization and, therefore, are assumed to contribute to different functional repertoires [24]:

Five subunits in the vermis: A, AX, X, B, and A2

Four subunits in the paravermis: C1, CX, C2, and C3

Three subunits in the hemispheres: D1, D0, and D2

### 2.3. Cerebellar Projections to the Cerebral Cortex

The cerebrocerebellar connectivity divides the cerebellar cortex into two sensorimotor and three nonmotor areas. The two sensorimotor areas include the I-VI rostral lobules and the VIII lobule. The three nonmotor areas include the VI caudal-Crus I lobules, the Crus II-VIIB lobules, and the IX-X lobules [15,25]. The Lobules I-VI rostral and VIII have dense connections with the primary motor cortex. In contrast, the lobules VI caudal-Crus I, Crus II-VIIB, and IX-X have reciprocal projections with the dorsolateral prefrontal cortex [26].

The three nonmotor areas process four “superiors” functions: working memory, language, social interaction, and management of emotions [15]. Working memory and social interaction are specifically represented only in the lobules VI caudal-Crus I and Crus II-VIIB. In contrast, language and management of emotions are broadly expressed in all three nonmotor areas [15].

A lesion in the lateral portion of the sensorimotor regions often causes dysmetria. Hypermetric movements are suggestive of a cerebellar deficit but are not limited to cerebellar lesions. Hypermetric movements appear following lesions of cerebellar efferents, for example, the thalamus [4].

In conclusion, these recent studies have shown the segregation of the motor and cognitive functions in the cerebrocerebellar system, the dentate nucleus showing also a somatotopy. Since the homogeneous crystal-like architecture characterizes the cerebellar cortex, a universal principle underlies the predictive computations in these motor and cognitive domains. Each PC in a microzone receives a massive number of mossy fiber-GC-mediated inputs and integrates motor efference copy and afferent inputs. These unique cerebellar architectures satisfy the basic requirements for the computation of an internal forward model. Microzones would store motor engrams thanks to PFs linking PCs to generate a coordinated behavior. 

## 3. Internal Models for Motor Control and Motor Learning

### 3.1. Internal Model for Delay Compensation

Unlike industrial robot control, biological motor systems face a unique problem that sensory feedback signals from peripheral receptors have an inevitable delay in reaching the sensory areas in the brain [27]. Therefore, the brain always observes the past state of the body and the world. Among a range of factors contributing to the sensory feedback delays, the nerve conduction delay dominates as reported in locomotor reflex movements of terrestrial animals, ranging 10 ms for a shrew to 100 ms for an elephant [28,29]. The sensory feedback delay imposes a restriction on specifically rapid movements in a fraction of a second, causing oscillatory and even unstable movements. Consider a feedback controller based on delayed sensory feedback to guide a hand to a target. The controller initially pushes the hand toward the target, and accordingly, the hand approaches the target. But when the hand reaches the goal, the controller still pushes the hand further away because the controller believes that the hand has not reached the destination yet. Therefore, the hand overshoots the target, resulting in a hypermetric movement. The controller then pulls the hand back toward the target, but again the correcting movement becomes hypermetric, i.e., passing the target toward the body. In this way, a feedback controller based on delayed feedback signals causes oscillatory movements. But animals, including humans, can perform rapid actions properly without overshooting or undershooting. There must be a control mechanism to compensate for delayed sensory feedback. 

**Figure 6:**
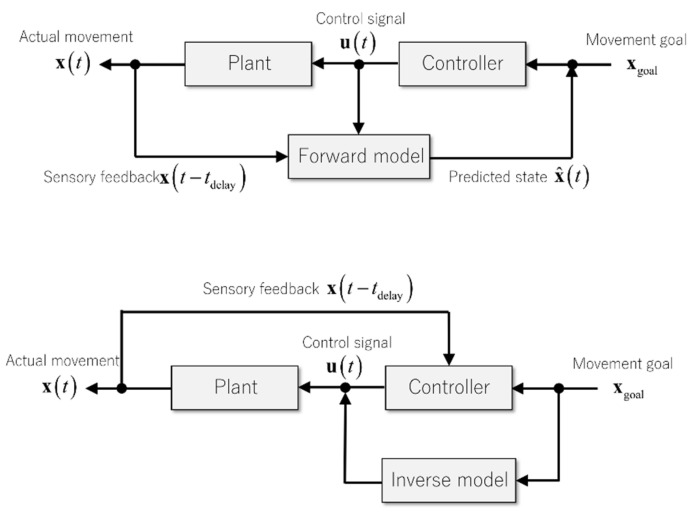
Computational diagrams of internal models. An internal forward model integrates sensory feedback signals and an efference copy of a control signal and predicts the current status of the body (top). In contrast, an internal inverse model produces control signals designed to achieve a given movement goal (bottom). Note that the outputs of a forward and an inverse model reside in the sensory and the motor coordinates, respectively.

One proposed solution is a mechanism called an internal model that emulates the dynamics of body and environments internally in the brain. There are, in general, two kinds of internal models depending on the ways of how the external dynamics realize in these models: an internal forward model and an internal inverse model [10] (Figure 6). An internal forward model (or forward model in short) solves the dynamics forward in time, essentially solving the equations of motion by combining the former state of the body from peripheral sensory organs and the efference copy from the motor area in the brain [9,30]. The predicted current state of the body from a forward model then drives a feedback controller to guide a movement. This type of controller is often termed as an internal feedback controller because not the actual sensory feedback signal, but the internal prediction from a forward model drives the feedback controller [31].

In contrast, an internal inverse model (or inverse model for short) transforms a predefined, desired trajectory into control signals by calculating the required force to realize the desired plan [32,33]. The control signals generated from an inverse model follows from the desired trajectory and does not depend on delayed sensory feedback signals. If the desired trajectory as an input to an inverse model is a function of time, a controller based on an inverse model is also a function of time instead of the body state. In control engineering, a controller that maps time to control signals is defined as feedforward, whereas a controller that maps a state to control signals is defined as feedback. According to the definitions, a controller based on an internal forward model is a feedback controller, and a controller based on an internal inverse model is a feedforward controller. In summary, both forward and inverse models solve the feedback-delay problem by incorporating the dynamics of controlled objects.

### 3.2. Internal Model for Motor Learning

Prediction of consequences caused by a motor action not only guides online motor control as reviewed above but also drives motor learning processes. Motor learning refers to a learning process in which a sensorimotor mapping from sensory inputs to motor outputs is acquired or revised according to experiences such as motor errors, rewards, or punishments. In motor learning, an actual consequence of a motor action is compared to an expected outcome of that action, leading to a prediction error. There, the prediction from a forward model assesses the performance of the actual action and then drives the learning process for further improvement. Lines of psychophysical studies support that prediction errors but not task errors contribute to a motor learning process [34,35,36]. The prediction-based motor learning parallels with reward-based reinforcement learning; a reward prediction error between an actual reward and an expected reward, but not a reward itself, facilitates a learning process [37,38]. Therefore, in biological learning processes, either error-based or reward-based, predictive computation plays a crucial role in assessing the current performance and its improvement.

### 3.3. Internal Model for Interlimb Coordination

In addition to delay compensation and motor adaptation, the predictive computation of a forward model plays a possible role in coordination among multiple degrees of freedom and in properly controlled timing in muscle activity onsets. All parts of the body are not independent of each other but interact dynamically, so controlling one degree of freedom requires the prediction of dynamical interactions from other degrees of freedom. In a ball-catching task, a subject first releases a ball from one hand and then catches the ball on a tray supported by the other hand. Healthy control subjects demonstrate electromyography (EMG) patterns in the catching hand that anticipates the timing of the ball contact on the tray [39,40]. This experiment suggests that the consequence of the movement of the releasing hand predicted from a forward model induces the predictive muscle activity in the catching hand. In contrast, cerebellar patients demonstrate EMG patterns only after the ball contact on the tray, suggesting an impairment of predictive computation of self-generated movements. As in the case of bimanual coordination, in multi-joint actions, a movement in one joint influences changes in other joints through interaction torques such as Coriolis and centrifugal forces. Therefore, coordinated and appropriately timed activities in multiple muscles necessitate the dynamic prediction of body states. 

In conclusion, the predictive computation of a forward model serves several computational functions: online state prediction for stable control of rapid movements, calculation of prediction errors in motor learning, and coordination and timing control among multiple degrees of freedom. Therefore, we anticipate that an impairment of the predictive computation would beget a multitude of symptoms. Also, an emergent view of the cerebellum illustrates that the cerebellum predicts not only the body state but also the expected reward. We will revisit the implication of a forward model and its impairment in later sections (*see* Section 5 and Section 6).

## 4. Patterns of Discharges in the Cerebellar Cortex

Neurophysiological analyses of PC activities provide a comprehensive way of understanding the predictive function of the cerebellum. The cerebellar PCs generate two different types of discharges, simple spikes, and complex spikes, originating from mossy-fiber inputs and climbing-fiber inputs, respectively [41]. These two action potentials are initiated in the proximal axon of PCs [42] and represent the only output of the cerebellar cortex to the deep cerebellar nuclei, providing powerful inhibitory signals [43,44]. According to the forward internal model, PC firings represent the predictions of the motor command consequences and the corresponding sensory feedback, allowing the cerebellum to correct the different parameters of movement [45]. We argue below that simple and complex spikes of Purkinje cells encode real-time prediction for online motor control and motor prediction error for motor learning, respectively.

### 4.1. Simple Spikes

Simple spike potential is unique depolarisation of PCs, at high frequency, varying from 0 to 250 Hz with an average of 50 Hz [44]. Mossy fibers conduct inputs to the cerebellum from the spinal, brainstem, cortical, and hypothalamic afferents [41,44]. Mossy fibers project into the cerebellar cortex and provide activating collaterals to the deep cerebellar nuclei, Golgi, and GCs. GCs modulate the activity of PCs though their axons and contact other distant PCs through PFs [43,44]. The massive convergence of inputs through ascendant axons and parallel fibers from GCs to PCs performs the integration of neural information from different cortical and peripheral sources. The multiple synapses between PFs and PCs, approximately 200,000 [41], suggest a complex combination of kinematic and error signals as well as a rich representation of motor behavior inside each cell [46]. This structural convergence confers to PCs the functional properties of a perceptron [47]. 

While simple spikes activities modulate weakly during passive movements and strongly during active movements [48], the pseudo-random tracking model reveals the role of simple spike firing in encoding the different parameters of arm movement, including position, velocity, and acceleration. Regression analyses based on each of these kinematic parameters found that the firing rates encoding individual parameters were mutually independent to each other and that velocity was the dominant parameter followed by position and speed [49,50]. Therefore, the simple spike activity of PCs plays a role in predicting the kinematic consequences of motor commands [49,51]. Moreover, the representation of arm kinematics was task-independent, meaning that a global limb model was used instead of large numbers of internal models for specific movements [46]. The random tracking paradigm also indicated a modulation of simple spike activity during error processing, especially during performance error, defined as the difference between the intended action and its consequences [46,49]. Therefore, PC simple spike discharges accounted for the computation of sensory prediction errors, namely the result of the cancellation between the prediction of the consequences of a motor command and the real-time sensory feedback of this command [46].

The analyses of temporal properties of simple spike modulation unravel a dual encoding property of individual PC [46]. The simple spike activity decreases when the action consequence is correctly predicted and increases when there is a discrepancy between the prediction and the outcomes [46].

### 4.2. Complex Spikes

Complex spikes have a highly stereotyped burst of decrementing spikes, whose rate (typically average 1 Hz) is modulated by the excitatory drive of climbing through large voltage-gated calcium conductance in the dendrites of PCs [52]. A single climbing-fiber input serves as a salient cell-wide signal recognized as a supervised signal. Complex spikes transiently inhibit simple spike firings of the same PC in two time scales: a short time scale (so-called post-complex spike pause) allowing the transmission of the teaching signal to cerebellar nuclei, and a long time scale where rates of complex spikes are anticorrelated with the rates of simple spikes in response to sensory stimulation. 

The encoding of error signals by the olivocerebellar tracts is necessary to instruct learning [53]. Climbing fiber activity, combined with preceding parallel-fiber inputs, induces long-term depression or long-term pootentiation of synaptic strengths of PF inputs to PCs depending on increase or decrease of climbing fiber activity (see pp.81-84 in [54]). And the synaptic plasticity serves as a neural substrate of cerebellum-driven motor learning [55]. Electrophysiological evidence suggests that complex spikes encode motor errors of voluntary arm movements [56] and eye movements [57]. The rate of complex spikes is higher in response to cues predictive of undesired successive stimuli, showing a contribution of the cerebellar cortex in the processing of aversive stimuli [58]. Moreover, recent data suggest that the cerebellum is also critically involved in reward mechanisms, especially the expected reward size [53]. Therefore, the roles of complex spikes are now expanding beyond the single purpose in motor learning. The motivational value is also intrinsic to human motor behavior. In either motor control or reward expectation, the cerebellum expects a consequence of its actions.

In conclusion, PC simple spikes encode kinematic parameters, including velocity, position, and acceleration in a random tracking task. Simultaneously these discharges account for the computation of predicting upcoming errors. Therefore, PCs can monitor the current state of the body by encoding kinematic parameters and prepare for a movement correction by calculating the expected errors. These properties of PC simple spikes enable rapid and stable control of the body and favor a view of the cerebellum as a locus of an internal forward model.

## 5. The Cerebellum as a Locus for Internal Forward Model

This section summarizes two of our recent studies that support the internal-forward-model hypothesis of the cerebellum. The first study compared behavioral data of healthy controls and ataxic patients in a wrist-tracking task and found that the predictive component of the movement showed increased error and delay compared to that of the controls. The second study analyzed single-unit activities of the cerebellar cells in a behaving monkey during a step-tracking movement of the wrist joint and found that the current output of the cerebellum (dentate cells) to cortical motor areas was predictive for the future input from the motor areas to the cerebellum (mossy fibers). 

### 5.1. Behavioral Evidence for the Internal-Model Hypothesis of the Cerebellum

**Figure 7:**
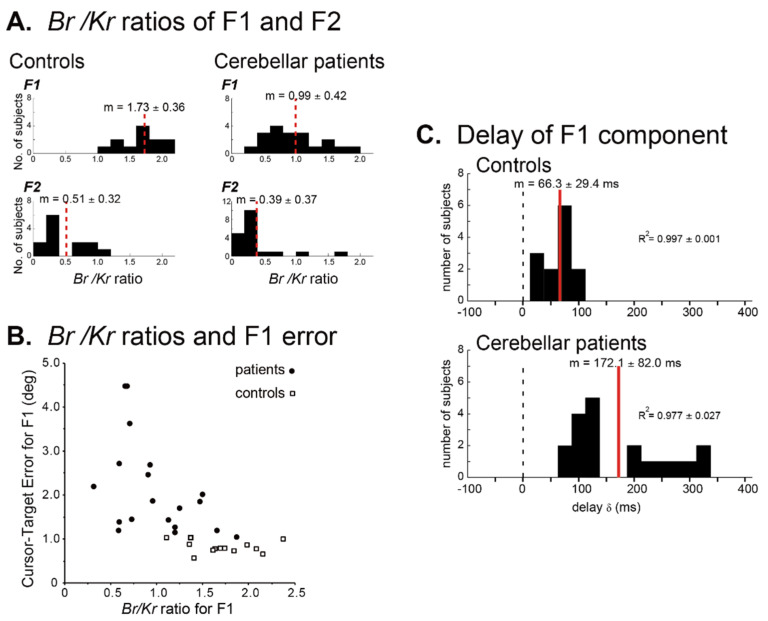
Difference of muscle activities and movement kinematics between controls and cerebellar patients. (**A**) Comparison of the Br/Kr ratios for the F1 and F2 components between the controls and the cerebellar patients. Controls: Br/Kr ratios of the control subjects for the F1 component (top) and the F2 component (bottom) (*n* = 13). Note the highly significant difference between the two components. Cerebellar patients: Br/Kr ratios of the patients for the F1 (top) and the F2 (bottom) components (*n* = 19). Note the selective decrease of Br/Kr ratios for the F1 component in the patients. (**B**) Correlation between the Br/Kr ratios for F1 component and Cursor-Target error for F1 (F1 error, in short). The Cursor-Target error for F1 (F1 error) is defined as an average error between the target motion and the F1 component of the movement during a trial. Note the negative correlation. (**C**) Delay of the predictive component of the movement (i.e., F1 component) relative to the target motion calculated with a cross-correlation analysis for Controls (*n* = 13) and Cerebellar patients (*n* = 19).

A series of studies from our group confirmed the impaired predictive component in dysmetric movements. There, we analyzed components of muscle activities in terms of the movement kinematics of the wrist joint in a smooth tracking task [59,60,61]. Specifically, we established a relationship between the muscle tension of prime wrist movers and movement kinematics (the wrist angle *θ*(*t*) and the wrist angular velocity $\dot{\theta }\left(t\right)$) weighted by the coefficients of *K_r_* (the elastic term) and *B_r_* (the viscous term) [59,60,61]. Importantly, we found that the ratio of *B_r_/K_r_* represented the recipe of muscle activities (i.e., motor command) in smooth pursuit movements. The muscle activities for the wrist movement were decomposed into a low-frequency (≤0.5 Hz) component (F1) and a high-frequency (>0.5 Hz) component (F2), each of which represented the predictive control and the feedback correction, respectively [62]. For the F1 component of the control subjects, the *B_r_/K_r_* ratio had a higher value (Figure 7A), suggesting the dominance of the velocity-dependent control. On the other hand, for the F2 component of the control subjects, the *B_r_/K_r_* ratio had a much smaller value (Figure 7A), indicating that this component represented intermittent correction of positional errors [62]. In contrast, patients with cerebellar ataxias (CAs) showed a selective decrease of the *B_r_/K_r_* ratio for the F1 component (Figure 7A), suggesting difficulty in recruitment of the predictive velocity control seen in the control subjects and dependence on the position-dependent corrections [62]. The loss of component-specific differences in the *B_r_/K_r_* ratio suggests impairment of predictive control in CAs. Indeed, the decrease in the *B_r_/K_r_* ratio correlated with the increase of error in the predictive movement (i.e., F1 component) (Figure 7B) [62]. Another critical difference between the control subjects and the patients with CAs was the increased delay of the F1 (predictive) component in the patients (Figure 7C). In the control subjects, the predictive movement (F1 component) lagged the target motion only by 66 ms, which was too small to be a visual feedback delay [62]. In contrast, in the patients with CAs, the same delay was increased by more than 100 ms, as much as 172 ms, and compatible with a visual feedback delay. Overall, our behavioral analysis elucidated the selective impairment in patients with CAs of predictive reproduction of the target velocity in terms of both accuracy and delay.

### 5.2. Neural Evidence for the Internal Forward Model Hypothesis of the Cerebellum

A theory in neuroscience should guide what an experiment must investigate and what consequences the experiment should yield to support or falsify the theory. One of the prime examples is the Marr-Albus-Ito theory of the cerebellar cortex; the prediction of plasticity in the synapses from parallel fibers to a Purkinje cell set the stage for the subsequent experimental endeavors [55]. In a similar vein, the theory of an internal forward model may provide a straightforward and experimentally confirmable prediction; the current output from an internal forward model should predict the future input to the model. If the cerebellum is to function as a forward model, the theory requires that the current cerebellar output (activities of dentate cells, DCs) should contain predictive information about the future cerebellar input (activities of mossy fibers, MFs). We, therefore, set out to examine neural activities recoded from the cerebellar cells covering MFs (cerebellar inputs), PCs (output from the cerebellar cortex), and DCs (cerebellar outputs) [63]. 

The dataset we analyzed contained activities of 94 MFs, 83 PCs, and 73 DCs recorded from a behaving monkey performing the wrist step-tracking task toward one of the eight targets [64,65]. We first examined how neural activities of one population were transformed into those of the next population. Given the feedforward connectivity of the cerebellum, we first attempted to reconstruct each PC’s firing rate as a linear weighted sum of those of 93 MFs. The linear prediction explained the activities of PCs excellently, where the median value of the goodness-of-fit was as high as 0.95. This success of the linear model was unexpected for two reasons. First, we used only 93 MFs for the reconstruction, but a typical PC receives parallel fiber inputs on the order of 10^5^. Second, there is a population of granule cells and Golgi cells between MFs and PCs, which may enable a complex nonlinear transformation of MF inputs. So, we expected that models with some nonlinearities could provide much better fitting for the activities of PCs. We then compared the linear model with a thresholded nonlinear model, a quadratic nonlinear model, and a finite-impulse model. A statistical comparison test revealed that the linear model explained the firing rate most parsimoniously. Subsequently, we proceeded to reconstruct the firing rates of DCs as a linear weighted sum of those of MFs and PCs, considering the anatomical connectivity that DCs receives inhibitory inputs from PCs and excitatory inputs from collaterals of MFs. The linear-model fitting was again most distinguished, where the median value of goodness-of-fit was as much as 0.94. Overall, our analysis revealed the linear transformation of cerebellar activities across populations. 

**Figure 8:**
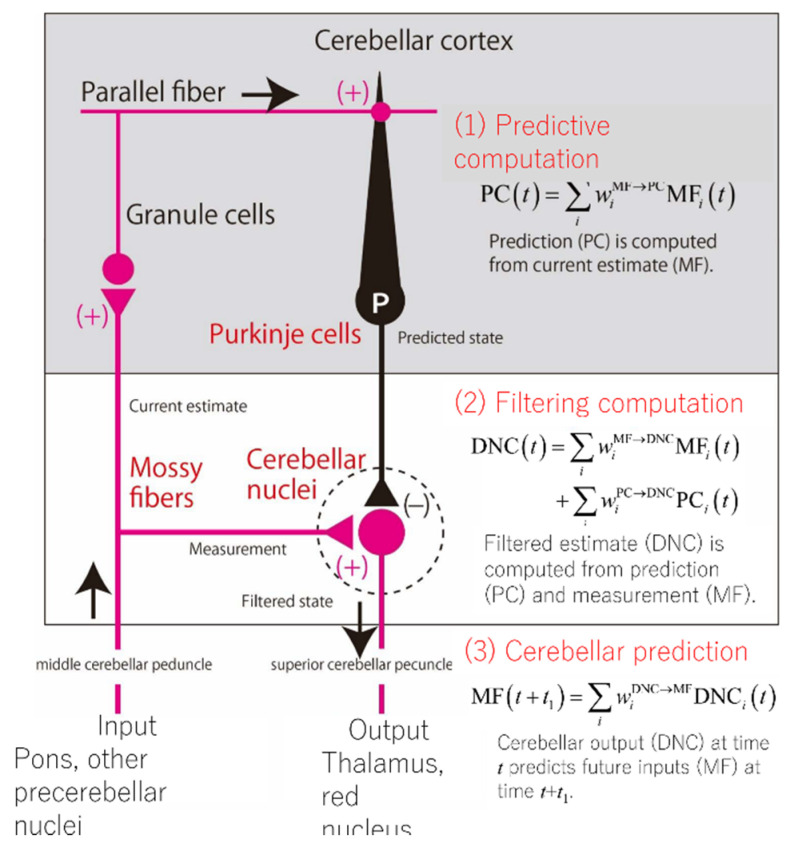
Schematics of the cerebellar circuit and corresponding computational steps. (**1**) Prediction computation. The PCs computes a predicted state from a current estimate conveyed by the mossy fibers (MFs). (**2**) Filtering computation. The predictive state computed by the PCs is integrated with an observation signal conveyed by other MFs for optimal estimation in DNCs. (**3**) Cerebellar prediction. The current output from the cerebellar circuit (DNCs) can predict future inputs to the cerebellum (MFs).

We finally tested the prediction of the internal-forward-model hypothesis of the cerebellum: the predictability of future cerebellar inputs from current cerebellar outputs. Expanding the linear analyses explained above, we fit a linear prediction model that reconstructed the firing rates of MFs at time *t* + *t*_1_ as a linear weighted sum of the firing rates of DCs at time *t*. The time advance *t*_1_ was a parameter to determine how much future the cerebellar output should predict the cerebellar input. When *t*_1_ ranged from 20 ms to 200 ms, the median goodness-of-fit decreased only moderately from 0.89 to 0.86. A statistical permutation test confirmed that these values were statistically significant, rejecting a null hypothesis that the linear prediction was caused just by a statistical coincidence. Our result of linear prediction affirms empirically that the cerebellum as a whole operates as an internal predictive model, which outputs a prediction of future inputs. We finally note that the linear relationship between the MF, PC, and DC activities resemble those of an optimal estimator known as the Kalman filter [62,65]. In the Kalman filter, there are two steps: the prediction step and the filtering step. By extending the analogy in the equations, we speculate that the MF-PC transformation corresponds to the prediction step and that the PC-, MF-DC transformation corresponds to the filtering step (Figure 8). 

In summary, our analysis of electrophysiological data provided direct evidence of the internal forward model, namely the prediction of a future outcome from a current state in the cerebellum [66].

## 6. Cerebellar Dysmetria: Kinematic and EMG Features

This section aims to revisit EMG and kinematic features associated with dysmetria. We argue the underlying pathophysiological mechanisms of dysmetria from the perspective of an internal forward model. 

### 6.1. EMG and Kinematic Features Associated with Dysmetria

The EMG and kinematic features of dysmetria have been analyzed using the task of fast goal-directed flexion movements [4,67,68,69,70,71,72,73]. Subjects (humans or monkeys) flex the elbow, wrist, or index finger as fast as they can by moving relatively heavy manipulators. Regular fast goal-directed flexion movements have a single-peaked velocity profile and triphasic activity in the pair of agonist and antagonist muscles. The triphasic muscle activity includes an initial agonist burst for generating an acceleratory pulse, the subsequent antagonist burst for providing a decelerator pulse, and the recurring agonist burst for achieving the accuracy toward the aimed target [68,74,75,76,77]. In ataxic patients and monkeys whose dentate nucleus becomes temporarily inactivated by cooling, hypermetria appears in both the proximal and distal joints. We below summarize the five characteristics found in the EMG and kinematic patterns associated with dysmetria [67,68,69,70];

(*1*) *Less phasic agonist activities*. The initiation of the initial agonist EMG burst shows delayed initiation, low rate of rise and small amplitude, and a prolonged duration [67,68,69,70].

(*2*) *Delayed antagonist activities.* The onset of antagonist EMG burst is delayed, and such a delay is sometimes associated with a low rate in the rise of EMG burst [67,68,69,70]. 

(*3*) *Skewnessin acceleration and deceleration.* The magnitude of the acceleration is decreased, but that of decelerations is increased [69,70].

(*4*) *Oscillations (tremor)*. The velocity profile shows more than one peaks [69].

(*5*) *Contrast features between oscillations and dysmetria.* Movements with oscillations reach the target with increased variability in the end-position, whereas movements without oscillations are often hypermetric. With an external load added to the manipulation, the movements change from being normetric with oscillations to being hypermetric without oscillations. An oscillation often appears in slower movements, whereas fast movements often show the sign of dysmetria [69].

### 6.2. Poor Prediction and Compensatory Feedback Corrections

The EMG and kinematic features reviewed above can originate from an impaired prediction of body and environment. Holmes argued the delayed onset of antagonistic activation as the core factor of hypermetria because the antagonistic activity brakes ongoing movements [78]. Previous studies showed that, in fast goal-directed movements, the EMG burst in the antagonist muscles has predictive natures of central origin; (*1*) onset before the movement [79], (*2*) amplitude independent of that of agonist EMG burst [80], (*3*) preserved burst even when the movement and a stretch in antagonist muscle are blocked [79], (*4*) reduced activity when the movement is mechanically terminated [81], (*5*) preserved activity in deafferentiated subjects by neuropathy [82], and (*6*) variable activity timing in ataxic patients under modified dynamics with an external load [72]. These findings conclude that the antagonist muscle activity reflects predictive control of a central origin [70]. 

A series of studies from our group has supported this predictive origin of muscle activity. We identified the receipt of motor commands in humans by analyzing the components of muscle activity in terms of movement kinematics of the wrist joint in smooth tracking task (*see* Section 5) [59,60,61,62]. In these malfunctions of predictive controllers, the ataxic patients had no choice but to rely on position-dependent step-wise corrections, which often caused a lack of movement smoothness. In this regard, such a compensatory feedback strategy could cause tremorous oscillations in the task of a fast goal-directed tracking movement. Consistently, a contrast relation was observed between oscillations and dysmetria [69,70] [*see the above* EMG and kinematic features (*5*)], i.e., when the trajectory was adjusted using oscillations, dysmetria became small. The compensatory feedback corrections in our recordings correspond with discontinuities in movements by Hore and Flament [83]. Discontinuities in movements occurred in an elbow flexion task, which was followed by a terminal tremor [83]. The transcortical loop is assumed to underlie both of them.

Importantly, the tremorous movements observed in ataxic patients are highly irregular and different from regular oscillations that are generated as an inherent problem in the reflex controller or as potentiation of a central oscillator [84,85]. For example, the cerebellum contributes to damp regular oscillations in a servo-control loop, since inactivation of the interpositus nucleus induces periodic oscillations [84]. Indeed, in a wave-variable processor model where the cerebellum is assumed to contribute to the servo-motor mechanism, a reduction in cerebellar output results in large regular oscillations [86]. Furthermore, dysmetria and tremor are independent of each other in essential tremor [87].

These behavioral observations indicate that EMG and kinematic features of the goal-directed tracking tasks in ataxic patients are related to the impairment of continuous predictive controls substituted by intermittent step-wise corrections. It should be noted that these step-wise corrections themselves are controlled with the impaired predictive controller and therefore result in irregular tremorous oscillations.

### 6.3. Algorithm of Predictive Control: Timing Versus Synergy?

Although there have been a number of studies suggesting that the cerebellum serves as a predictive controller [62], the predictive parameters used in the controller are not clear. Clinical observations and physiological experiments suggest two candidates for the predictive control targets, timing, and synergy. On the other hand, recent experimental evidence suggests that the PC-dentate nucleus (DN) neuron pathway [64] generates fine-tuned temporal patterns of output using disinhibition/inhibition modes. Thus, it is necessary to examine whether the impairment of the disinhibition/inhibition modes can elicit disturbances in the timing and/or synergy control. 

#### 6.3.1. Timing

Holmes proposed that impaired movements in the nose-finger test are attributed to the delay in initiation (asthenia) and deficit in postural fixation (adventitious movements) [78,88]. He also described that “in addition to regulating postural tone, the cerebellum reinforces or tunes up the cerebral motor apparatus, including subcortical structures with motor functions, so that they respond promptly to volitional stimuli and the impulses from them which excite muscular contractions are properly graded” [88]. Holmes’ proposal of timing control by the cerebellum can be traced back to Luciani (1915), who postulated that the cerebellum serves to exert a supportive influence on the rest of the nervous system, namely cerebellar fine-tuning of motor control [89,90]. These seminal studies derived the timing theory based on observations of single-joint movements. It follows that irregularities in movements of multiple joints are the results of errors in constituent single-joint movements [90]. Overall, accumulating evidence has confirmed the involvement of the cerebellum in precise timing control [91]. Patients with CAs show a disproportionate increase in temporal variability during a finger-tapping task compared to a circle drawing task, suggesting ataxic impairment in movement timing [92]. They also show disrupted timing in conditioned eyeblink responses in the eyeblink conditioning test [93]. Thus, there is a general agreement that the cerebellum plays an essential role in the predictive control of timing in triphasic EMG patterns, especially in antagonist bursts [91]. 

#### 6.3.2. Synergy

In parallel with the timing-control theory of the cerebellum, another theory stresses the coordinative function of the cerebellum, which we refer to as the synergy-control theory. Thach [94] emphasized that “the cerebellum combines and integrates the many factors to initiate coordinated movements of the many body parts, and it thus enables to play the unique role of initiating coordinated movements”. The cerebellar role in coordinating movements becomes more critical in multi-joint movements. This idea originates from a series of classic work published more than a century ago [94]. Babinski [95] defined synergy as “an association of movements that constitute a single act”. Consistently, inactivation or ablation of the cerebellar nucleus did not affect simple movements. On the other hand, compound movements were impaired and eliminated [96]. A recent study showed that a PC inhibited and disinhibited an activity of a selective muscle, but not in surrounding muscles, allowing execution of movement performed with a small number of muscles [97]. These findings point to an impairment in coordinating multiple degrees of freedom or synergy. Besides, Thach (2014) argued that in a reaching task, the cerebellum organizes compound movements based on the physical properties of body parts, including muscle strength, segment inertia, joint viscosity, and segmental interaction [94].

A study on neuronal activities in monkeys showed that a vast majority of PCs, with somatosensory receptive fields (RFs) in the forearm, was suppressed strongly before the onset of wrist movements. In contrast, a vast majority of DNs with the same RFs showed a concurrent increase in the activity [64]. The central commands for the agonist bursts of wrist muscles are assumed to be under intense modulations by cerebellar outputs (via the cerebello-thalamo-cortical pathways) [64]. Thus these observations suggest that activation of DNs generated by reduced inhibition from PCs, i.e., disinhibition, facilitates the execution of wrist movement. On the other hand, suppression of the DNs by increased PC activity contributes to the stabilization of proximal muscles that are not directly involved in the wrist movements themselves. 

Based on these neural mechanisms, altered timing and amplitude of disinhibition will provide not only delayed timing of activities of agonist and/or antagonist muscles (i.e., change in timing), but also reduced rates of the rise with altered amplitudes (i.e., change in synergy). In fact, noninvasive cerebellar stimulation reinforced activities of the chained inhibitory neurons, resulting in improvement in the relative timing of agonist/antagonist bursts [98]. On the other hand, the disinhibition/inhibition of activities of a particular muscle can be considered from a synergic context of agonist/antagonist bursts. For instance, insufficient braking activity of the triceps muscle during elbow flexion could be attributed to altered synergy that encodes for simultaneous inhibition of the flexor muscles (agonist muscles) and disinhibition of the extensor muscles (antagonist muscles).

Despite their apparent differences, the timing theory and the synergy theory could be two aspects of an impairment of the predictive control using the internal forward model. It should be emphasized that even a synergic control requires adjustments of both timing and amplitude of individual muscle activities. Taken together, we believe that the cerebellum contributes prediction of proper temporal and spatial parameters of the body and its environment for coordinated compound movements and that both timing and synergy are essential aspects of the prediction (*see* Section 3).

### 6.4. Dysmetria Spanning Multitudes of Time Scales

**Figure 9:**
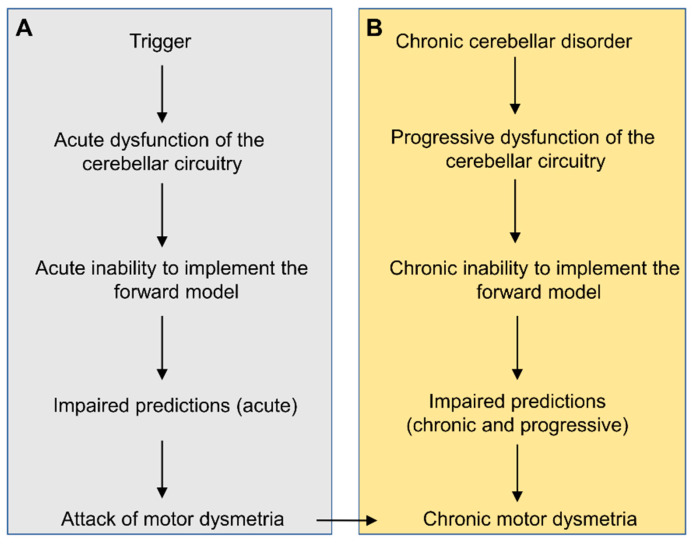
General framework on the mechanism of motor dysmetria in episodic ataxias (**A**) and in chronic cerebellar ataxias (**B**). (**A**): attacks of ataxia may be triggered by external events (exercise, emotions, or startle) in a context of channelopathy. The triggers induce an acute dysfunction of the cerebellar circuitry, resulting in impaired forward models causing motor ataxia. (**B**): in chronic cerebellar disorders, the degenerative process affecting the cerebellar cortex and/or the cerebellar nuclei leads to chronic dysfunction of microcircuits. Predictions cannot be computed accurately.

An impairment of the predictive computation explains a range of characteristics manifested in cerebellar ataxia, as reviewed above. The finding that the prediction of the internal forward model underlies not only compensation of sensory delay but also motor learning indicates that the internal forward model administers motor control and learning in multiple time scales. In this regard, a theory of the internal forward model could provide a comprehensive framework for dysmetria not only for episodic ataxias but also for chronic and permanent ataxias. The attacks of ataxia would represent recurrent impairments in the update/building of the internal model. Figure 9 summarizes the critical roles of motor predictions in the pathogenesis of motor dysmetria, both in attacks of episodic ataxias and in chronic cerebellar disorders.

In conclusion, EMG and kinematic features associated with dysmetria can be explained without contradiction by inappropriate timing and synergy controls in cerebellar prediction. 

## 7. General Conclusions

This review article proposes that the prediction of the internal forward model is critical both for motor control and learning and that the neural circuitry of the cerebellum is befitting for implementing the neural computation of the internal-forward-model prediction. The two recent studies from our group support the forward-model hypothesis of the cerebellum from behavioral and neurophysiological perspectives. Recent progress in clinical neurophysiology evinces that dysmetria in cerebellar motor ataxias is a result of impairments in an internal forward model embedded in the cerebellum. Although there have been historical arguments whether dysmetria can be attributed to deficits in timing or impediments in synergy, timing anomalies and asynergy could be two aspects of an elementary impairment of the predictive control using the internal forward model. The cerebellum forms reciprocal connections with both (a) the cortical motor areas and the prefrontal areas and (b) basal ganglia, indicating that the cerebellum coordinates with those cortical areas for motor and non-motor functions. The uniform anatomical circuit in the cerebellum suggests that the predictive computation in the cerebrocerebellum contributes to not only motor controls but also to non-motor, so-called ‘cognitive’ functions, not only for episodic ataxias but also for chronic ataxias. We expect that future studies will advance our understanding of the predictive nature and its impairment underlying dysmetria in the cerebellar cognitive affective syndrome (Schmahmann’s syndrome).
